# Gene profiling of head mesoderm in early zebrafish development: insights into the evolution of cranial mesoderm

**DOI:** 10.1186/s13227-019-0128-3

**Published:** 2019-07-06

**Authors:** Huijia Wang, Peter W. H. Holland, Tokiharu Takahashi

**Affiliations:** 1 Faculty of Biology, Medicine and Health, The University of Manchester, Oxford Road, Manchester, M13 9PT UK; 2 Department of Zoology, University of Oxford, Zoology Research and Administration Building, 11a Mansfield Road, Oxford, OX1 3SZ UK

**Keywords:** Head mesoderm, Cranial paraxial mesoderm, Cranial lateral mesoderm, Pharyngeal mesoderm, Anterior lateral plate mesoderm, Cardiopharyngeal field, Amphioxus, Zebrafish, Head segmentation

## Abstract

**Background:**

The evolution of the head was one of the key events that marked the transition from invertebrates to vertebrates. With the emergence of structures such as eyes and jaws, vertebrates evolved an active and predatory life style and radiated into diversity of large-bodied animals. These organs are moved by cranial muscles that derive embryologically from head mesoderm. Compared with other embryonic components of the head, such as placodes and cranial neural crest cells, our understanding of cranial mesoderm is limited and is restricted to few species.

**Results:**

Here, we report the expression patterns of key genes in zebrafish head mesoderm at very early developmental stages. Apart from a basic anterior–posterior axis marked by a combination of *pitx2* and *tbx1* expression, we find that most gene expression patterns are poorly conserved between zebrafish and chick, suggesting fewer developmental constraints imposed than in trunk mesoderm. Interestingly, the gene expression patterns clearly show the early establishment of medial–lateral compartmentalisation in zebrafish head mesoderm, comprising a wide medial zone flanked by two narrower strips.

**Conclusions:**

In zebrafish head mesoderm, there is no clear molecular regionalisation along the anteroposterior axis as previously reported in chick embryos. In contrast, the medial–lateral regionalisation is formed at early developmental stages. These patterns correspond to the distinction between paraxial mesoderm and lateral plate mesoderm in the trunk, suggesting a common groundplan for patterning head and trunk mesoderm. By comparison of these expression patterns to that of amphioxus homologues, we argue for an evolutionary link between zebrafish head mesoderm and amphioxus anteriormost somites.

**Electronic supplementary material:**

The online version of this article (10.1186/s13227-019-0128-3) contains supplementary material, which is available to authorized users.

## Background

The head is one of the most elaborate structures in the vertebrate body, formed through the combination and interaction of cells with different embryonic origins. Several of the characteristic structures of the head, such as the jaws, facial bones and special sense organs, are derived embryologically from cranial neural crest cells (CNCs) and epidermal placodes. Both CNCs and placodes are regarded as unique to vertebrates (although putative precursors, without the same developmental fates, have been identified in urochordates, the sister group to vertebrates [1–3]). Consequently, the vertebrate head has been argued to be an evolutionarily novel structure, with the emergence of the ‘New Head’ core to vertebrate origins and evolution [4]. In contrast, the muscles and braincase in the craniofacial region are mostly derived from head mesoderm, not from CNCs or placodes. Mesoderm has a very long evolutionary history, dating back to the origin of bilaterian animals or even before [5] and is not specific to vertebrates. The New Head hypothesis [4] did not argue that whole vertebrate head was a brand new structure in evolution, and mesoderm can be considered an ‘old part’ of the ‘New Head’. Nonetheless, head mesoderm has undergone its own evolutionary transformations. Mesodermal tissues in the vertebrate head and body are organised quite differently. To understand the evolution of the vertebrate head, therefore, we must consider not just CNCs and placodes, but also the major organisational changes that took place in mesoderm.

The head mesoderm of vertebrates comprises different mesodermal populations, namely prechordal, cranial paraxial and (at least in some vertebrates) cranial lateral mesoderm (also called the pharyngeal mesoderm) [6]. At an early stage of development, the prechordal mesoderm is located anterior (rostral) to the notochord and beneath the neural tube and is also called prechordal plate. This region of mesoderm is unique in being unpaired and axial. The cranial paraxial mesoderm is, in terms of position, the anteriormost part of the paraxial mesoderm that lies in two strips along the anterior–posterior axis of the entire vertebrate body, on either side of the notochord. However, while paraxial mesoderm is divided into transient repeated units (somites) in most of the body, these are not obvious in the cranial paraxial mesoderm. Instead, in the head region the paraxial mesoderm forms a solid block of cells either side of the notochord. The evolutionary origin of this difference between head and trunk has been hotly debated and is unresolved [6]. Cranial lateral mesoderm lies more laterally and ventrally, close to the pharynx, but is not clearly distinguished by any morphological boundary at early stages of development. As development proceeds, the prechordal mesoderm is displaced caudally and laterally by growth of the anterior neural plate and subsumed into the rest of the cranial mesoderm.

In vertebrate embryos that have been extensively studied, the head mesoderm is not clearly segmented. This is in contrast to the trunk region of vertebrates where the paraxial mesoderm is segmented to form somites. In amphioxus, a marine invertebrate animal closely related to vertebrates, the paraxial mesoderm shows clear segmentation all the way from head to tail. This contrast between amphioxus and vertebrates creates a conundrum. Is the amphioxus condition secondarily modified, with an unsegmented anterior region having evolved into a segmented region, perhaps by co-option of cyclical patterning mechanisms? Or was the head mesoderm originally segmented in the ancestor of vertebrates [7, 8]? If the latter, traces of cryptic segmentation might still be detectable in the cranial mesoderm of living vertebrates. Three lines of evidence have been proposed in favour of such cryptic segmentation, although confusingly each favours a different number of segments. First, scanning electron microscopy suggested the presence of either four or seven subtle pairs of ‘whorls’ of mesenchymal cells in cranial mesoderm [9–12], although these findings have been controversial and difficult to replicate. Second, waves of cyclical gene expression associated with somitic segmentation suggest possibly two repeated units in cranial mesoderm of chick embryos [13, 14]. And third, classical histological examination of elasmobranch embryos, such as sharks and rays, detects three clear epithelial cavities in cranial mesoderm, a finding long regarded as reflecting original segmentation in vertebrate head [15, 16]. These head cavities are not detected in all vertebrates, however, and recent molecular studies have revealed gene expression similarities to cranial mesoderm of amniotes rather than to shark somites, suggesting they may be a secondary peculiarity and not a reflection of ancestral segmentation [17]. In summary, it is still unclear whether the cranial mesoderm of vertebrates evolved from a segmented state and how it may relate to segmentation in amphioxus.

Additional clues to cranial mesoderm origins could come from considering its intrinsic regionalisation. Studies on the early subdivision of the vertebrate head mesoderm have been dominated by research using bird embryos, including a long series of classic transplantation experiments using quail-chick chimeras and labelling studies using DiI labelling or retroviruses to trace cell fates [18–22]. More recently, the molecular subdivision of the head mesoderm prior to the onset of differentiation has been studied in detail [23]. The restricted expression of genes such as *Pitx2* in the anterior and *Tbx1* in the posterior region reveals that anterior–posterior regionalisation does exist in the morphologically uniform head mesoderm, although the gene expression boundaries for these markers are imprecise and expression patterns overlap [24]. Indications of regionalisation in the chick head mesoderm are further shown by expression and functional analysis of genes encoding components of signalling cascades, notably members of the retinoic acid (RA), bone morphogenetic protein (BMP) and fibroblast growth factor (FGF) pathways [25].

Thus far, investigations into gene expression in vertebrate head mesoderm development have been largely limited to amniotes (such as birds and mammals), plus more recently elasmobranchs and lamprey. In contrast, there have been fewer studies into the molecular development of the cranial mesoderm of teleost fish, particularly as might be relevant to understanding regionalisation or segmentation. Genes have been reported to be expressed in zebrafish head mesoderm, but these have not been studied comparatively from the view point of evolution and development. Particularly in zebrafish, the anterior lateral plate mesoderm (ALPM) has been extensively examined in studies focused on haematopoietic, endothelial and cardiogenic cells [26, 27]; however, the relationship between ALPM and cranial lateral mesoderm is not clearly resolved.

In this study, we examined the spatial expression of genes encoding transcription factors and molecules involved in signalling cascade in the zebrafish head mesoderm with particular focus on early developmental stages. We use these data to discuss differences and similarities between the head mesoderm between zebrafish, other vertebrates and amphioxus and to address the evolution of the vertebrate head mesoderm.

## Methods

### Animals

Zebrafish (*Danio rerio*) were kept under standard laboratory conditions at 28.5 °C in a 12/12 h light/dark cycle in the fish facility at the University of Manchester, UK. The zebrafish AB line was used for obtaining wild-type embryos. After collecting embryos from natural mating, they were kept in E3 medium (5 mM NaCl, 0.17 mM KCl, 0.33 mM CaCl2, 0.33 mM MgSO4, 0.0001% methylene blue) at 28.5 °C. Embryos after 75% epiboly (75E) were staged as hours post-fertilisation (hpf) or as somite stages (ss) [28]. After reaching the target developmental stages (8hpf/75E, 10hpf/bud, 11hpf/3ss, 11.67hpf/5ss, 12.5hpf/7ss, 13.5hpf/9ss and 15hpf/12ss), zebrafish embryos were dechorionated manually and fixed in 4% paraformaldehyde (PFA) in 1X phosphate-buffered saline (PBS) overnight at 4 °C, followed by dehydration in 25%, 50%, 75% of methanol in PBS, and were kept in 100% methanol at -20 °C until use.

### In situ hybridisation

Whole-mount in situ hybridisation (WISH) was performed using digoxigenin (DIG)-labelled RNA probes as previously described [29]. Before hybridisation, embryos were rehydrated in PBS. Zebrafish total RNA was isolated from bud stage embryos using TRIzol reagent (Invitrogen), and the template cDNA used for PCR was prepared from extracted RNA using SuperScript III First-Strand Synthesis SuperMix (Invitrogen). For the preparation of RNA probes, DNA fragments of marker genes (*gsc*, *pitx2*, *isl1*, *foxc1a*, *fsta, tbx1*, *cyp26c1*, *alx1* and *tbx20* with sizes 750 to 994 bp; Additional file 1) were amplified by RT-PCR and cloned into the pGEM-T Easy Vector (Promega). DIG-labelled RNA probes were synthesised by in vitro transcription from linearised plasmids except for *fsta* and *cyp26c1*, which were generated directly from PCR products using T7 RNA polymerase [30]. The *pitx2* probe used in this study can detect *pitx2a* and *pixt2c* isoforms [31]. For imaging, embryos were re-fixed in 4% PFA/PBST (1X PBS with 0.1% Tween-20), mounted in glycerol and imaged using a Leica DFC 7000T digital camera attached to a Leica M165 FC compound microscope. All images were processed by Leica Application Suite X.

### Multi-probe fluorescence in situ hybridisation

Triple-colour in situ hybridisation was conducted using in situ hybridization chain reaction (HCR) v3.0 technology [32]. DNA probe sets for zebrafish *foxc1a*, *tbx1* and *tbx20* genes were generated by Molecular Instruments, Inc (CA, USA). In situ HCR was performed following the standard protocol for zebrafish embryos [33] except that the probe concentration was increased to 10 nM. Zebrafish embryos fixed at 3ss were used in the experiment without proteinase K treatment. Each mRNA signal was amplified and labelled by Alexa Fluor 546 for *foxc1a*, Alexa Fluor 647 for *tbx1* and Alexa Fluor 488 for *tbx20,* respectively.

### Confocal microscopy

A Leica TCS SP8 AOBS inverted confocal microscope equipped with a 40X/1.30 oil objective and 0.75X confocal zoom was used to image whole-mount zebrafish embryos and sections. Each embryo was carefully embedded in 1% agarose gel before imaging. The confocal settings were: pinhole 1AU, scan speed 400 Hz unidirectional, format 512X512. Images were collected using HyD detectors with the following detection mirror settings: Alexa Fluor 488, 494-540 nm; Alexa Fluor 546, 561-640 nm; Alexa Fluor 647, 657-747 nm using the white light laser with 490 nm (25%), 556 nm (30%) and 650 nm (25%) laser lines, respectively. All images were collected sequentially. When acquiring 3D optical stacks, the confocal software was used to determine the optimal number of Z sections. Only the maximum intensity projections of these 3D stacks are shown in the results.

### Cryosection of zebrafish embryos

After WISH, stained embryos were rinsed in 1X PBS and immersed in 15% sucrose/PBS at room temperature for 1.5 h. Embryos were then transferred into 20% gelatin/15% sucrose overnight. Embryos were embedded in a mould containing 20% gelatin and left on dry ice for 2 h before being transferred to the cryostat. Blocks were sectioned at 12 μm thickness and collected onto Superfrost Plus (VWR international) slides. Sections were mounted under a glass coverslip in ProLong Diamond Antifade Mountant with DAPI (Invitrogen).

## Results

Relatively few genes have been reported with spatial expression in cranial mesoderm of vertebrates or the anterior somites of amphioxus. We selected nine genes: seven encoding putative transcription factors and two encoding components of cell–cell signalling pathways. Although information exists concerning expression of several of these in zebrafish, our goal was to obtain detailed descriptions of spatio-temporal gene expression in parallel and enable accurate comparison to expression patterns reported for other vertebrates and amphioxus.

### Gene expression in anterior head mesoderm at early developmental stages

We first consider gene expression in the prechordal mesoderm of zebrafish. Prechordal mesoderm cells are derived from anterior hypoblast (mesendoderm) during zebrafish gastrulation [28] and undergo migration as a group of cells from the germ ring margin towards the animal pole [34]. The most anterior part of the prechordal plate forms a prominent bulge called the polster at the bud stage occurring at 10 h post-fertilisation (10hpf).

#### goosecoid

**Fig. 1:**
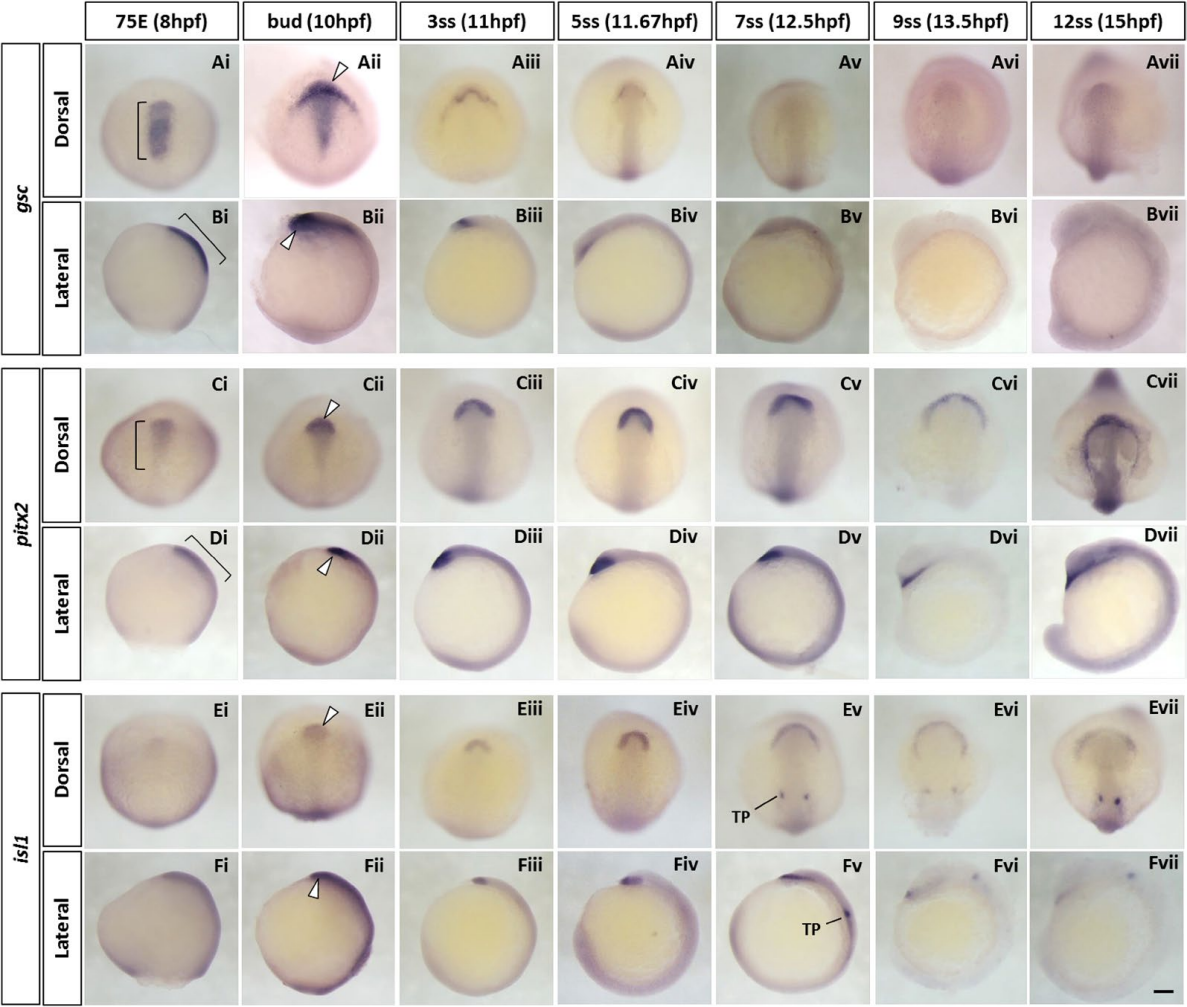
Spatio-temporal expression of prechordal plate marker genes (*gsc*, *pitx2* and *isl1*) during zebrafish early development. **A**–**F** Whole-mount in situ hybridisation of zebrafish prechordal plate/polster marker genes. Embryos were hybridised with *gsc* **(Ai–vii, Bi–vii)**, *pitx2* **(Ci–vii, Di–vii)** and *isl1* probes **(Ei–vii, Fi–vii)**. Expression of all three marker genes is detected in the pre-polster, a distinct group of cells located underneath the forebrain, at 75% epiboly stage **(Ai, Ci, Ei)**. *gsc* and *pitx2* transcripts are also detected in the posterior prechordal plate during early development **(Ai–ii, Bi–ii, Ci–ii, Di–ii)**. From 7-somite stage onwards, expression of *isl1* was also seen in the trigeminal placodes (**Ev-vii, Fv-vii**). White arrowheads indicate the expression of marker genes in the polster, the most anterior part of the prechordal plate. Brackets indicate strong expression of *gsc* **(Ai, Bi)** and graded expression of *pitx2* from the anterior to the posterior tip in the prechordal plate during late gastrula period **(Ci, Di)**. In all images, anterior is oriented to the top. Lateral images **(B, D, F)** are viewed from the left side of the embryos. 75E, 75% epiboly; hpf, hours post-fertilisation; ss, somite stage; TP, trigeminal placode. Scale bar: 100 μm

The zebrafish *goosecoid* (*gsc*) homeobox gene is a long established marker for the prechordal plate [35, 36]. At the mid-gastrula stage (Fig. 1Ai, Bi), the anterior hypoblast migrating towards the animal pole shows clear *gsc* expression. At the bud stage, strong *gsc* expression continues in a broad anterior crescent-shape (the polster) as well as in more posterior parts of the prechordal plate in the midline (Fig. 1Aii, Bii). Over the next hour to the 3-somite stage (3ss), the latter expression reduces dramatically (Fig. 1Aiii, Biii), whereas expression in the polster decreases more gradually and is detectable until 12ss (Fig. 1Avii, Bvii).

#### pitx2

The *pitx2* homeobox gene is one of the downstream targets of *gsc* in the prechordal plate [37]. At the 75% epiboly stage (75E), the *pitx2* gene is already expressed in the prechordal plate (Fig. 1Ci, Di) and becomes consolidated to the most anterior regions from the bud stage onwards (Fig. 1Cii, Dii), with clear and strong expression becoming limited to the polster from 3ss onwards (Fig. 1Ciii–v, Diii–v). From the 9ss (Fig. 1Cvi–vii, Dvi–vii), the polster forms a U-shaped mesendodermal structure in front of the boundary of the anterior neural plate [38]. In contrast to reported expression of *Pitx2* in chick embryos [24], we detect no zebrafish *pitx2* expression in the cranial paraxial mesoderm at any developmental stages examined.

#### islet1

*islet1* (*isl1*) is a marker of the cardiac progenitors in the secondary heart field [39, 40] but is also expressed in the anterior prechordal plate [41]. We detect weak expression of *isl1* in the most anterior prechordal region around 75E (Fig. 1Ei, Fi) with the signal becoming stronger and localising to the U-shaped polster over next few hours (Fig. 1Eii–iv, Fii–iv). Like *pitx2* gene, *isl1* mRNA expression is not detected in cranial paraxial mesoderm; unlike *pitx2*, the *isl1* expression signal fades markedly by 12ss (Fig. 1Ev–vii, Fv–vii).

### Gene expression pattern in zebrafish head mesoderm in three bilateral strips

In zebrafish, the paraxial mesendoderm derives from bilateral territories either side of the shield [42, 43]. It develops by convergent extension during gastrulation [34], and its most anterior part becomes the cranial paraxial mesoderm [44].

#### foxc1a

Zebrafish *foxc1a* and *foxc1b* are duplicate genes homologous to amniote *Foxc1*, a member of the forkhead box transcription factor family. Zebrafish *foxc1a* is reported to be expressed in the presomitic mesoderm, developing somites, adaxial cells and head mesoderm [45]. Mouse *Foxc1* (formerly known as *Mf1*) and chick *Foxc1* (reported as *cFKH*-*1*) are also expressed in the cranial paraxial mesoderm [46, 47]. Interestingly, distribution of both zebrafish and mouse *foxc1a*/*Foxc1* is graded along the mediolateral axis, with highest levels closest to the midline.

**Fig. 2:**
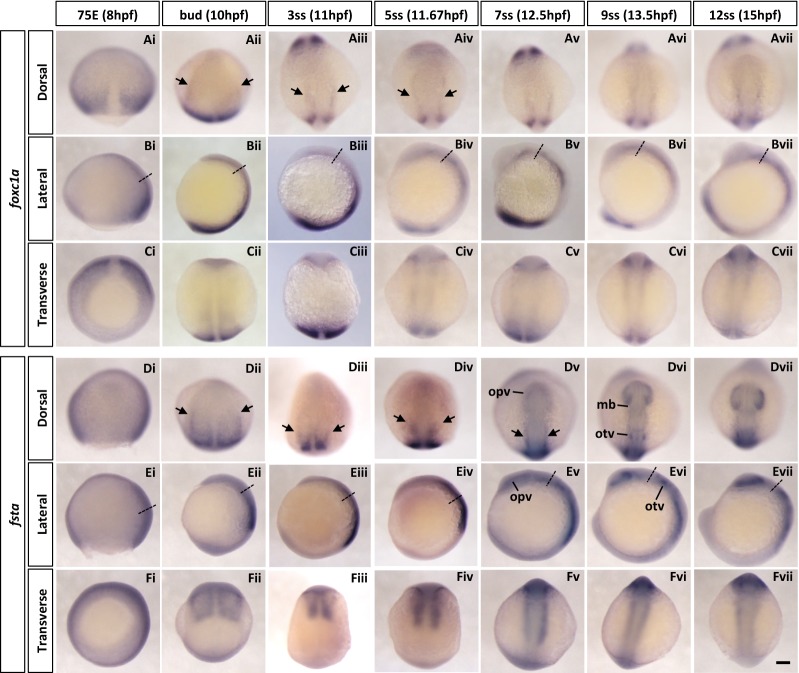
Spatio-temporal expression of cranial paraxial mesoderm marker genes (*foxc1a* and *fsta*) during zebrafish early development. **A**–**F** Whole-mount in situ hybridisation of zebrafish cranial paraxial mesoderm marker genes. The embryos were stained with *foxc1a* **(Ai–vii, Bi–vii, Ci–vii)** and *fsta* **(Di–vii, Ei–vii, Fi–vii)** probes. Both genes were expressed in the paraxial mesoderm, including the cranial paraxial mesoderm and the presomitic mesoderm. Black arrows indicate the cranial paraxial mesoderm. Anterior is to the top in dorsal and lateral views; in all transverse views, dorsal side is oriented to the top. Transverse views were taken at the level indicated by the dashed lines in lateral views. 75E, 75% epiboly; hpf, hours post-fertilisation; mb, midbrain; opv, optic vesicle; otv, otic vesicle; ss, somite stage. Scale bar: 100 μm

We observed that during gastrulation, *foxc1a* is expressed in the involuting paraxial mesoderm (Fig. 2Ai–ii, Bi–ii, Ci–ii). Expression in cranial paraxial mesoderm adjacent to the notochord is observed from 3ss onwards (Fig. 2Aiii–iv, arrows). *foxc1a* continues to be expressed in bilateral strips immediately next to the notochord as the embryo develops (Fig. 2Av–vii, Bv–vii, Cv–vii). More posteriorly, *foxc1a* expression in the presomitic mesoderm, newly formed somites and adaxial cells is also detected.

#### follistatin A

Zebrafish *follistatin A* (*fsta*) is one of the two follistatin genes homologous to amphibian *Follistatin* [48] encoding a Spemann organiser-specific secreted antagonist of BMP2/4. In zebrafish, *fsta* has the same dorsalising property as the *Xenopus* homologue, but is not reported to be expressed in the organiser region [49].

In our analysis, *fsta* mRNA is detected at the bud stage in the paraxial mesoderm (Fig. 2Dii, Eii). As embryogenesis progresses, expression domain of *fsta* in the cranial paraxial mesoderm becomes more constricted (Fig. 2Dii–iv, arrows). In addition to the mesoderm, *fsta* is expressed in the optic vesicles, the midbrain and the otic vesicles at later stages (Fig. 2Dv–vii).

#### tbx1

The *tbx1* gene encodes a member of the large family of T-box transcription factors. In humans, hemizygous loss of *Tbx1* has been suggested to be a major contributor to the cardiovascular/pharyngeal defects in DiGeorge syndrome, with similar phenotypes evident in mouse mutants [50–52] and the zebrafish *van gogh* mutant (*vgo*; caused by *tbx1* mutation) [53]. *vgo* also shows the lack of branchiomeric muscles that are derived from pharyngeal mesoderm.

**Fig. 3:**
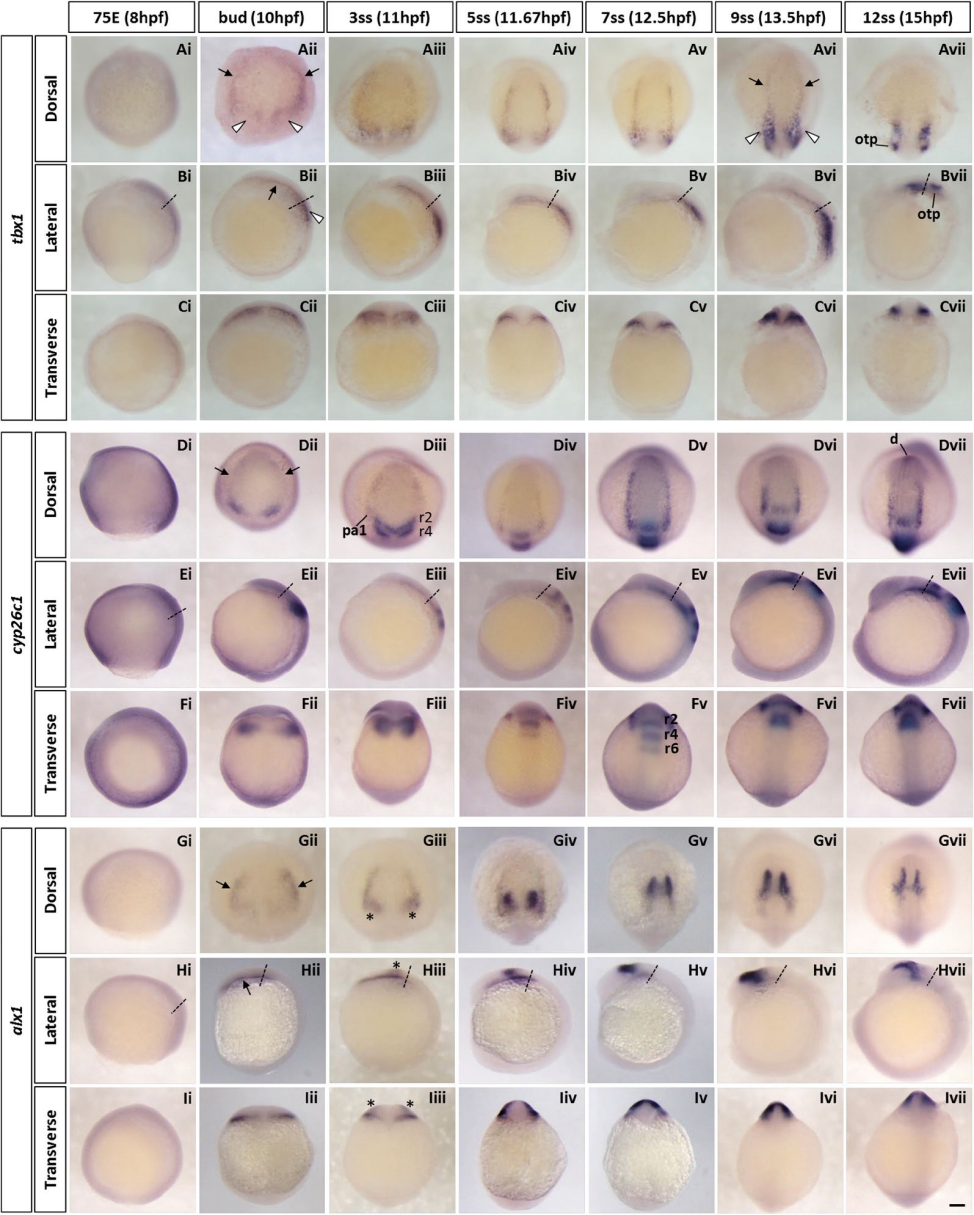
Spatio-temporal expression of cranial lateral mesoderm marker genes (*tbx1*, *cyp26c1* and *alx1*) during zebrafish early development. **A**–**I** Whole-mount in situ hybridisation of zebrafish cranial lateral mesoderm marker genes. Embryos were hybridised with *tbx1* **(Ai–vii, Bi–vii, Ci–vii)**, *cyp26c1* **(Di–vii, Ei–vii, Fi–vii)** and *alx1* **(Gi–vii, Hi–vii, Ii–vii)** probes. Expression of all three genes in the cranial lateral mesoderm can be seen from bud- to 5-somite stage (**Aii–iv, Dii–iv, Gii–iv**, arrows). Black arrows indicate the cranial lateral mesoderm marked by the three genes. White arrowheads show the *tbx1*-expressing posterior region in the cranial paraxial mesoderm. Asterisks highlight the neural crest population labelled with *alx1*. In dorsal and lateral views, anterior is oriented to the top; in transverse views, dorsal side is oriented to the top. Transverse views were taken at the level indicated by the dashed lines in lateral views. 75E, 75% epiboly; d, diencephalon; hpf, hours post-fertilisation; otp, otic placode; r, rhombomere; ss, somite stage. Scale bar: 100 μm

In the head mesoderm of early zebrafish embryos, *tbx1* shows an interesting expression pattern. From the end of gastrulation at the bud stage to 7ss, *tbx1* is expressed in bilateral strips (Fig. 3Aii–v, Bii–v, black arrows) along the lateral borders of the cranial paraxial mesoderm region expressing *foxc1a* and *fsta* in the anterior head region. Posteriorly, the medial border of the *tbx1*-expressing region extends medially towards the lateral edge of the notochord (Fig. 3Aii–v, Bii–v, white arrowheads). As the embryo develops, the bilateral strips move inward (Fig. 3Avi, black arrows) and the signal of *tbx1* expression at the posterior region is intensified with its shape becoming more elongated longitudinally (Fig. 3Avi, white arrowheads). At 12ss, *tbx1* is mainly expressed in this region and the anterior bilateral *tbx1*-strips disappear (Fig. 3Avii, Bvii). An additional expression domain is detected in the otic placodes at this stage.

#### cyp26c1

*cyp26c1* is one of three zebrafish paralogues encoding Cyp26 enzymes. Cyp26 enzymes are members of the P450 family proteins and control RA signalling by promoting the degradation of RA [54]. In chick embryos, *Cyp26c1* is expressed in the anterior cranial paraxial mesoderm from early development (Hamburger–Hamilton stage [HH] 5) and involved in the formation of the primary anterior–posterior axis in the head mesoderm [25].

As shown in Fig. 3Dii, zebrafish *cyp26c1* is expressed from the bud stage onwards, at the lateral margin (black arrows in Fig. 3Dii–iii) of the cranial paraxial mesoderm where *foxc1a* is expressed, as a pair of bilateral two strips as previously described (formerly named as *cyp26d1*) [55]. These *cyp26c1*-expressing longitudinal regions are located in the same position as the *tbx1*-expressing strips until 7ss (Fig. 3Dii–v); the bilateral strips move inward at later stages (Fig. 3Dvi–vii). A stronger expression is also observed around presumptive rhombomere 2/4 (Fig. 3Dii) and in rhombomere 2, 4 and 6 from 3ss onwards (Fig. 3Diii–vii, Fiii–vii).

#### alx1

*alx1* is a member of the Alx homeobox gene family [56]. In chick, a paralogous gene, *Alx4*, was demonstrated to be a marker gene of anterior cranial paraxial mesoderm [24]. We previously cloned and examined the expression of *alx4a* and *alx4b*, the zebrafish orthologues of chick *Alx4*, but found they were not expressed in the head mesoderm at early developmental stages [57]. Later *alx4a* and *alx4b* are expressed in the mandible and the hyoid arch, respectively.

In contrast to *alx4a/b*, we found that *alx1* is expressed in the lateral margin of the cranial paraxial mesoderm (Fig. 3Gii–iv, Hii–iv, Iii–iv) where *tbx1* and *cyp26c1* are expressed. From 3ss onwards, a second cell population expressing *alx1* is also detected, marking a region medial, dorsal and posterior to the cranial mesoderm bilateral strips; we previously showed [57] that these are CNCs derived from the midbrain (Fig. 3Giii, Hiii, Iiii, asterisks). The expression of *alx1* in the two strips of cranial mesoderm becomes weaker and eventually diminished by 9ss while the signal in CNCs becomes stronger and migrates forward (Fig. 3Giii–vii, Hii–vii). Later at 48hpf, *alx1* is expressed in the distal end of the mandible arch [57].

#### tbx20

**Fig. 4:**
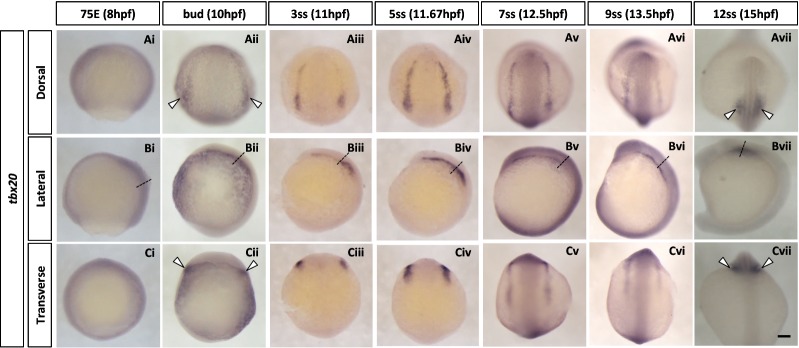
Spatio-temporal expression of anterior lateral plate mesoderm (ALPM) marker *tbx20* during zebrafish early development. **A**–**C** Whole-mount in situ hybridisation of zebrafish ALPM marker gene. Embryos were hybridised with *tbx20* **(Ai–vii, Bi–vii, Ci–vii)** probes. *tbx20* transcript can be first observed around bud stage in a reverse U-shaped domain **(Aii)**. This domain covers the anterior lateral plate mesoderm. Transverse views **(Ci–vii)** were taken at the level shown by the dashed lines illustrated in lateral images **(Bi–vii)**. Anterior is to the top in dorsal and lateral views. In all transverse views, dorsal side is oriented to the top. White arrowheads indicate the prospective heart primordium. 75E, 75% epiboly; hpf, hours post-fertilisation; ss, somite stage. Scale bar: 100 μm

*tbx20* encodes a T-box transcription factor reported to be expressed in the ALPM [58] and encodes an essential regulator of embryonic heart growth in zebrafish [59]. In chick embryos, *Tbx20* is expressed in the lateral plate mesoderm at early developmental stages and subsequently in primitive heart tube [60, 61]. We first detect expression of *tbx20* at the bud stage as a pair of arched strips (Fig. 4Aii). These bilateral strips are situated outside the lateral margin of the longitudinal expression sites of *tbx1*, *cyp26c1* and *alx1*. Posteriorly *tbx20* mRNA is expressed in the paired future heart primordium (Fig. 4Aii, Cii, white arrowheads). This expression pattern persists until around 9ss (Fig. 4Aii–vi, Bii–vi). As development proceeds, *tbx20* transcripts become more restricted to the heart primordium (Fig. 4Avii, Cvii, white arrowheads).

**Fig. 5:**
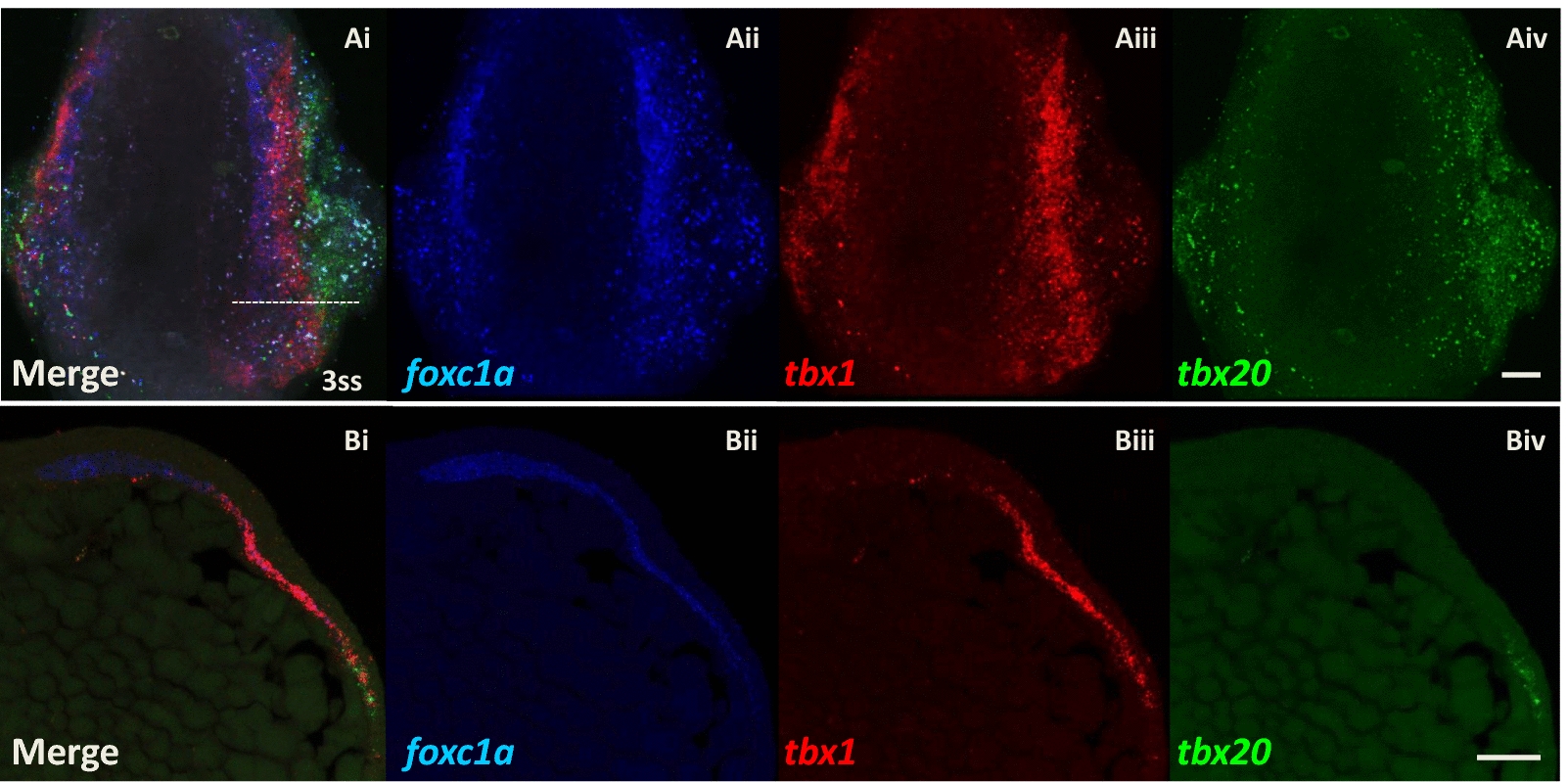
Expression patterns of *foxc1a*, *tbx1* and *tbx20* genes resolve into three longitudinal strips in zebrafish head mesoderm (3ss, 11hpf). Confocal microscope images of triple in situ hybridisation of *foxc1a* (dark blue), *tbx1* (red) and *tbx20* (green) mRNA. **Ai–iv** Dorsal views of a whole-mount zebrafish embryo at 3ss (11hpf), anterior is oriented to the top. Note that the embryo was slightly tilted towards to the left when embedded in agarose gel to show better the separation of expression regions on the right side of the embryo. The innermost paraxial strip (dark blue) expresses *foxc1a* gene, then a more lateral strip (red) expresses *tbx1*, and finally, the most lateral strip (green) expresses *tbx20*. The boundaries of the three regions overlap. **Bi–iv** Cross sections of a zebrafish embryo at 3ss showing the right side of the head mesoderm, dorsal is oriented to the left-top. The level of sectioning is at the anterior hindbrain shown as the dotted line in Ai. Scale bar: 50 μm

From the comparison of the above data based on single-probe WISH, the expression pattern of these marker genes appears to form three bilateral strips in the cranial mesoderm of zebrafish embryos at early developmental stages; an inner most strip marked by *foxc1a* and *fsta*, a middle strip by *tbx1*, *cyp26c1* and *alx1*, and the most lateral by *tbx20*. In order to scrutinise the positional relationship between these three expression domains, triple-colour in situ hybridisation was conducted in zebrafish embryos at 3ss. Figure 5 clearly shows that *foxc1a* (dark blue; Aii, Bii), *tbx1* (red; Aiii, Biii) and *tbx20* (green; Aiv, Biv) genes are expressed in three side-by-side longitudinal strips (Ai, Bi). The boundaries of the three regions partially overlap, and nonetheless, there is a clear mediolateral regionalisation in zebrafish head mesoderm at 3ss.

## Discussion

In this study, we examined the expression of key genes in zebrafish head mesoderm at early developmental stages. Expression of genes encoding transcription factors and components of signalling pathways have previously been analysed in detail in chick embryos [24, 25] and shark head mesoderm [17]. Comparing these taxa facilitates insights into the development and evolution of vertebrate head mesoderm.

### Conservation and divergence in vertebrate head mesoderm evolution

Posterior to the head, in the body or trunk region, gene expression patterns are remarkably congruent between vertebrate classes. Furthermore, apart from Hox genes, there is little molecular regionalisation along the anteroposterior axis of the somitic mesoderm. In contrast, previous work has shown the head mesoderm has clear molecular regionalisation along the anteroposterior axis prior to differentiation in chick embryos. Bothe and Dietrich [24] showed that the anterior region of the chick head mesoderm is marked by the expression of *Pitx2*, *Alx4*, *MyoR*, and the posterior part of head mesoderm by *Tbx1* and *Twist* genes. In the current study of zebrafish cranial development, we also find the most anterior region of the head mesoderm (prechordal plate) strongly expresses the *pitx2* gene during early development, anterior to a (cranial lateral) mesoderm region expressing *tbx1*. Therefore, a basic *Pitx2* anterior/*Tbx1* posterior axis is conserved between chick [24], shark [17] and zebrafish head mesoderm.

There are, however, some differences between species. Although zebrafish *pitx2* is strongly expressed in the most anterior part of the head mesoderm, its expression is restricted to prechordal mesoderm coincident with markers such as *gsc* [35] and *isl1* [62]. Unlike chick *Pitx2*, zebrafish *pitx2* is not expressed in the cranial paraxial mesoderm marked by *foxc1a* throughout the development (Fig. 1C, D). *Alx4* is another anterior marker of chick head mesoderm [24], but we find that the zebrafish orthologues, *alx4a* and *alx4b*, are not expressed in the head mesoderm [57]. Instead, a paralogous gene, zebrafish *alx1*, is expressed in cranial mesoderm, at the lateral margin of the paraxial region (Fig. 3Gii–iv). A third difference concerns *cyp26c1*, encoding a key enzyme for establishment of the RA anteroposterior gradient, which in chick is extensively expressed in anterior cranial mesoderm but in zebrafish is restricted to the lateral margin like *alx1* (Fig. 3Dii–v).

It is difficult to correlate precisely the developmental processes underpinning head mesoderm formation between different vertebrate classes since the mechanics of axis formation, developmental timing and topological relations between embryonic components differ, especially at early developmental stages. Even so, the differences in expression patterns of genes encoding transcription factors and signalling pathways between amniotes and teleosts suggest there are fewer developmental constraints imposed within the vertebrate head mesoderm than the trunk mesoderm. This may reflect versatility of head mesoderm to adapt to environmental differences, alongside diversification of craniofacial structures in evolution, and may be related to absence of overt mesodermal segmentation. Furthermore, this versatility may serve to make inference of ancestral gene expressions difficult.

### Mediolateral cranial regionalisation reveals similarities to trunk mesoderm

**Fig. 6:**
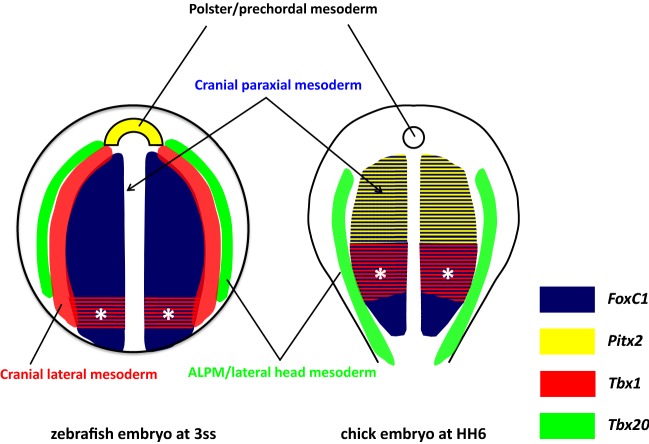
Schematic illustration of zebrafish cranial mesoderm pattering at early development stage (3ss) in comparison with chick embryo (HH6). Dorsal view of a zebrafish embryo at 3ss and the rostral region of a chick embryo at HH6. By the end of gastrulation, a distinct group of cells form the polster which lies at most anterior part of the prechordal plate mesoderm. These cells are marked by the expression of *pitx2* (yellow, left) in zebrafish but not in chick. Posterior to the polster, the zebrafish cranial mesoderm (left) resolves into three bilateral strips along the mediolateral axis. The innermost region is the cranial paraxial mesoderm (dark blue) located adjacent to the notochord and marked by the expressions of *foxc1a* and *fsta*; lateral to the paraxial mesoderm is the cranial lateral mesoderm (red) marked by the expression of *tbx1*, *cyp26c1* and *alx1* genes; the outermost strip is the anterior lateral plate mesoderm (ALPM, green) labelled with *tbx20*. Note that *tbx1* gene is also expressed in the posterior region (*) of the cranial paraxial mesoderm. The boundaries between the three mediolateral strips partially overlap. In contrast to zebrafish embryos, chick head mesoderm lacks the intermediate strip (red) of cranial lateral mesoderm. Instead, the paraxial mesoderm marked by *FoxC1* (dark blue) is further sub-regionalised into the anterior part marked by *Pitx2* (yellow/blue stripes) and posterior (*) by *Tbx1* (red/blue stripes). Chick *Tbx20* (green) is expressed in both anterior and posterior lateral mesoderm at this stage

Our results show clear compartmentalisation of the head mesoderm along the medial–lateral axis in early developmental stages of zebrafish. Expression patterns of marker genes resolve into three parallel bilateral zones or longitudinal strips (Fig. 5Ai–iv, summarised in Fig. 6): a broad innermost paraxial strip (Fig. 5Aii, dark blue area in Fig. 6) on each side immediately adjacent to the notochord and marked by *foxc1a* and *fsta* (and *tbx1* in its most posterior region, asterisks in Fig. 6), then a more lateral bilateral strip (Fig. 5Aiii, red stripes in Fig. 6) expressing *tbx1*, *cyp26c1* and *alx1*, and finally the most lateral pair of strips (Fig. 5Aiv, green stripes in Fig. 6) expressing *tbx20*. The last region has been established as the ALPM which later gives rise to blood cells, vascular cells and cardiac muscle progenitors [58]. The spatial expression patterns of *fsta* (paraxial) and *cyp26c1* (lateral) suggest that BMP and/or RA signalling may be involved in the establishment of this mediolateral regionalisation.

As development proceeds and the zebrafish embryo becomes elongated, these three pairs of bilateral strips shift inward and become squeezed. Indeed, from middle segmentation stages around 9ss, the strips are no longer clearly separated. Thus, the expression patterns described are visible only for a limited time window between 10hpf and 12.5hpf. The existence of a distinct central region (red stripes in Fig. 6), expressing *tbx1*, *cyp26c1* and *alx1,* between the cranial paraxial mesoderm and the ALPM is consistent with a previous report showing that *cyp26c1* is expressed medial to the ALPM (marked by *nkx2.5*-expression) with partial overlap at a slightly later stage [63]. Similarly, a recent live imaging analysis using *tbx1* reporter transgenic lines showed that the *tbx1*-expressing domain is medial to *draculin* (*drl*)-expressing ALPM cells, with some overlap [64]. Taken together, these data and our result (Fig. 5) reveal medial–lateral regionalisation in zebrafish head mesoderm for a limited time window at early developmental stages. In particular, in addition to the cranial paraxial mesoderm marked by *foxc1a* and the ALPM marked by *tbx20*, there is an intermediate transitional zone marked by *tbx1*. We suggest this region represents the cranial lateral mesoderm previously proposed [6]. The cardiopharyngeal field [65] may include both the ALPM and the cranial lateral mesoderm or could be separated into cardio-(*tbx20*-expressing) and pharyngeal-(*alx1/tbx1*-expressing) regions.

It is possible that the mediolateral regionalisation of the cranial mesoderm is comparable to the distinction between paraxial (somitic) and lateral plate mesoderm in the trunk region of vertebrates. This view conforms to the anatomical concept originally proposed by van Wijhe [66] based on observation of shark embryos (see also Kuratani and Adachi [67]). Such a view, however, would contrast with arguments that amniote head mesoderm does not have lateral plate or visceral structural components exclusive of cardiovascular tissue [68]. With regard to this issue, it is worth revisiting early transplantation experiments using quail-chick chimeras that traced cell fate in the head mesoderm [18]. In this study, Couly et al. showed that the cranial paraxial mesoderm in 3ss avian embryos can be separated in two longitudinal regions, medial and lateral to the notochord. The medial cranial mesodermal region was shown to give rise to extraocular muscles, whereas the lateral region yields branchiomeric muscles, a finding in line with van Wijhe’s view and our zebrafish early embryo data. However, in experiments using later stage embryos (HH9-10, 7-10ss), the mediolateral regionalisation was rearranged to a rostrocaudal sequence; that is, the anterior part of the head mesoderm gave rise to extraocular muscles and the posterior to branchiomeric muscles [69], possibly due to the migration and change of position of myogenic precursors. Insight into how branchiomeric-fated cranial cells relate to the molecular zonation comes from recent tracing studies using transgenic reporter lines in zebrafish. Live imaging analysis traced *tbx1* reporter-expressing cells from the cranial lateral mesoderm/ALPM and demonstrated that they migrate to the pharyngeal arches and to the heart by 36hpf [64]. Similarly, *drl*-expressing cells in the anterior region are characterised to give rise to blood cells, vascular cells and the second heart field as well as branchial muscles [70]. Therefore, both gene expression analysis and cell fate studies converge on the view that there are clear medial and lateral regions of zebrafish (and possibly chick) head mesoderm primarily established at early stages and that these can be usefully compared to paraxial (somitic) and lateral plate mesoderm of the trunk. From this point of view, the paraxial mesoderm is continuous from head to tail in early zebrafish embryos, suggestive of a common evolutionary origin for head and trunk mesoderm.

### Evolutionary origin of vertebrate head mesoderm

**Fig. 7:**
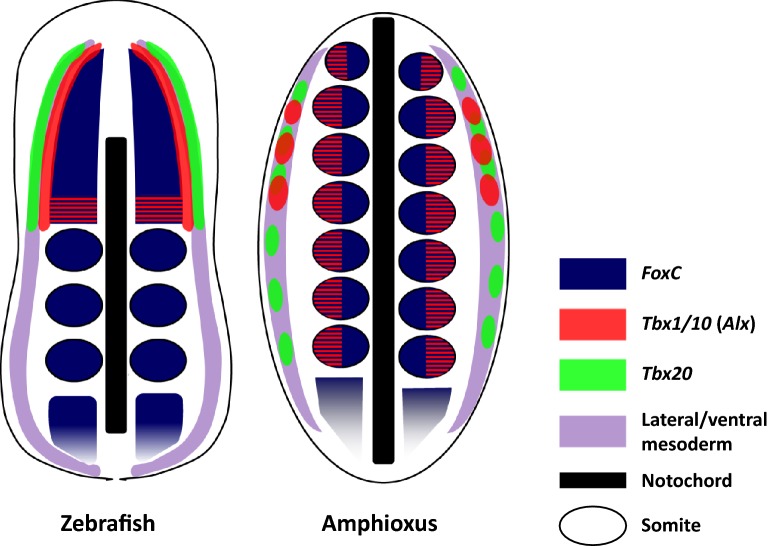
Comparison of the three-strip expression patterns of homologous genes between zebrafish and amphioxus embryos. Dorsal view of a zebrafish embryo at early segmentation period (3ss) and an amphioxus embryo at mid-neurula (7ss). In zebrafish head mesoderm at this developmental stage, expression patterns of marker genes resolve into three parallel bilateral strips. The innermost region marked by *foxc1a* (dark blue) expression continues to the posterior paraxial mesoderm which is segmented by somites. In the amphioxus embryo, *FoxC* is expressed in the somites all along the body. Lateral to the paraxial mesoderm in zebrafish is the cranial lateral mesoderm (red) marked by the expression of *tbx1*, *cyp26c1* and *alx1* genes. In the amphioxus embryo, *Tbx1/10* expression spans paraxial (somites) and ventral mesoderm. The latter is known to be homologous to vertebrate lateral mesoderm (wisteria), and *Tbx1/10* expression here is limited to the most anterior pharyngeal region. In the somites, *Tbx1/10* is expressed only in the ventral half of all the somites shown as red/blue stripes. *Alx* gene shows a similar expression pattern except that its somite expression is limited to the anteriormost region. In amphioxus, all three *Cyp26* genes are expressed only in the most anterior somites, but not in the ventral mesoderm. The outermost strip is the anterior lateral plate mesoderm (ALPM, green) labelled with *tbx20*. In the amphioxus embryo, *Tbx20* is expressed in the ventral mesoderm from anterior to posterior region. Note that the prechordal plate is not shown in this diagram

To understand the evolutionary origin of mediolateral regionalisation of zebrafish head mesoderm, it is useful to compare our data with gene expression patterns in amphioxus mesoderm (summarised in Fig. 7). In making these comparisons, it is worth noting that studies have already shown that ventral mesoderm of amphioxus is homologous to lateral mesoderm of vertebrates (shown as wisteria-coloured areas in Fig. 7), at least in the trunk region [71–73], despite differences in developmental timing and morphogenesis, and similarly that amphioxus somites have molecular similarities to vertebrate trunk somites [74–76].

Paraxial mesoderm in both cranial and trunk regions is marked by *foxc1a/b* in zebrafish ([45]; this study) and by *Foxc1* in chick and mouse [46, 47]. Similarly, the amphioxus homologous gene *FoxC* is expressed in the somites all along the body by mid-late neurulae (dark blue areas in Fig. 7) [77]. With regard to the outermost lateral mesoderm of zebrafish, *tbx20* ([58]; this study) and *nkx2*.5 [78] are established ALPM markers implicated in cardiac development; the amphioxus orthologues *Tbx20* [79] and *AmphiNk2*-*tin* [72, 73] are expressed in the ventral mesoderm (green in Fig. 7). In our study, the mesoderm lying between the cranial paraxial mesoderm and ALPM is marked by the combination of *tbx1* (Fig. 3A), *cyp26c1* (Fig. 3D) and *alx1* (Fig. 3G) expression. Two homologous amphioxus genes, *Tbx1/10* [80] and *Alx* [81], are expressed in the ventral half of somites and in more ventral mesoderm (red areas in Fig. 7); amphioxus *Cyp26* genes are expressed in somites but not in the ventral mesoderm [82]. Interestingly, the expression of the amphioxus homologues (*Tbx1/10*, *Alx*, *Cyp26*) is limited to mesoderm in the anterior part of the body.

Thus far, the middle region of the three strips has been confirmed visually only in zebrafish embryos. Although a quail-chick transplantation study [18] indicates the existence of a homologous region in chick embryos at 3ss, chick *Tbx1* expression has not been detected in the cranial lateral mesoderm at a comparable developmental stage [25]. Interestingly, *Xenopus Tbx1* shows a dynamic expression pattern reminiscent of zebrafish *tbx1*, moving from the paraxial/lateral mesoderm boundary to more posterior pharyngeal region [83], which suggests the ‘three-strip’ pattern of zebrafish head mesoderm is likely to reflect the ancestral condition for vertebrates (or at least Osteichthyes). This mediolateral compartmentalisation is detectable only in a very limited time window, and the detailed expression study at early developmental stages of vertebrates from outside the Osteichthyes, such as dogfish, will be informative to infer the head mesoderm organisation of vertebrate ancestors.

In summary, the three bilateral strips of gene expression in zebrafish head mesoderm are remarkably similar to the expression patterns of three sets of homologous amphioxus genes, staggered from paraxial to ventral in the anterior mesoderm of the body. This argues that the separation of paraxial and lateral mesoderm, and possibly that of somatic and visceral derivatives, was most likely already established in the common chordate ancestor. Furthermore, these distinctions extended to the ‘head’ end of the body, which deploys a distinct set of pattering genes to the more posterior or trunk regions.

These data do not reveal whether the common ancestor of amphioxus and vertebrates, the ancestor of all living chordates, had mesodermal segmentation in the head region. However, they do emphasise remarkable molecular similarities between the anteriormost (segmented) mesoderm of amphioxus and the cranial (unsegmented) mesoderm of vertebrates. These two regions are almost certainly homologous. We argue that vertebrate cranial mesoderm is not a novelty, nor an evolutionary transformation of a single segment. Instead, either the vertebrate head mesoderm evolved from an extensive segmented region by loss of somitic boundaries, or the anteriormost amphioxus somites were secondarily imposed during evolution on an extensive unsegmented mesodermal region.

## Conclusions

The expression patterns of key genes in zebrafish head mesoderm have some differences from those of other vertebrate species previously reported. Unlike chick embryos, there is no clear anteroposterior regionalisation in the cranial paraxial mesoderm of zebrafish. In contrast, medial–lateral compartmentalisation in zebrafish head mesoderm is established at early developmental stages, as indicated by expression patterns of three sets of marker genes resolving into three bilateral longitudinal strips. This mediolateral regionalisation of the cranial mesoderm is comparable to the distinction between paraxial and lateral plate mesoderm in the trunk, suggesting that head and trunk mesoderm of vertebrates share a common evolutionary origin.

Moreover, the three bilateral strips of gene expression in zebrafish head mesoderm are comparable to the expression patterns of three sets of homologous amphioxus genes. Considering the data together, we argue that the separation of paraxial mesoderm and lateral mesoderm was already established in the common chordate ancestor prior to the separation of head and trunk in vertebrates, suggesting that the anteriormost somites of amphioxus and the cranial mesoderm of vertebrates are homologous.

## Additional file

**Additional file 1.** List of the PCR primers used in this study and NCBI ID for genes investigated.

## Data Availability

All data generated during this study are included in this published article.
