# Supplementary material for: Gene profiling of head mesoderm in early zebrafish development: insights into the evolution of cranial mesoderm
Source: EvoDevo. 2019 Jul 6;10:14. doi: 10.1186/s13227-019-0128-3 (PMC6612195; doi:10.1186/s13227-019-0128-3)
Supplement: Supplementary file 1 — Additional file 1. List of the PCR primers used in this study and NCBI ID for genes investigated. [file 13227_2019_128_MOESM1_ESM.docx]

List of the PCR primers used in this study and NCBI ID for genes investigated

| **gene** | **PCR primer sequences** | | **NCBI Reference** |
| --- | --- | --- | --- |
| *gsc* | Forward:  Reverse: | 5’-CGCCGGCTCTGTGCTTATTT-3’  5’-TGTTCCTGTTTTCAGGCGAC-3’ | NM_131017.1 |
| *pitx2* | Forward:  Reverse: | 5’-TCCTTGCTCTCGGCTGAAAC-3’  5’-GGGCTGCATAAGTCCGTTGA-3’ | NM_130975.2 |
| *isl1* | Forward:  Reverse: | 5’-CCAGCCTGCTTTCCAACAAC-3’  5’-TGCATGCTTAGTACTTGGGC-3’ | NM_130962.1 |
| *foxc1a* | Forward:  Reverse: | 5’-TTTACTACCCCGTGGTGGAC -3’  5’-CGTCTGACGCATTTCAACAC-3’ | NM_131728.3 |
| *fsta* | Forward:  Reverse: | 5’-TTTTGCGCTGCTCGTCTATC-3’  5’-GCCGGGAATTTAATACGACTCACTATAGGGCTCCAGGAGAACCCCCAAAG-3’ | NM_131037.3 |
| *tbx1* | Forward:  Reverse: | 5’-GCCAACACGCACGAGTTTAG-3’  5’-TCTGCCATTGGGTCCATTCC-3’ | NM_183339.1 |
| *cyp26c1* | Forward:  Reverse: | 5’-ATCATCCTGGGACCCAACAC-3’  5’-GCCGGGAATTTAATACGACTCACTATAGGGATAAGCTTCTGCCGTCTCGT-3’ | NM_001029951.2 |
| *alx1* | Forward:  Reverse: | 5’-TTGAGACGAGGCCAGAGGAC-3’  5’-CCTGGCTCTGTGAATAATTACAAG-3’ | NM_001045074.1 |
| *tbx20* | Forward:  Reverse: | 5’-TCCATAGCCGCACTCATGTC-3’  5’-GCAAACGGGTTGCTGTCAAT-3’ | NM_131506.2 |
